# Psychosocial Impacts of Huntington's Disease on Individuals, Relatives and Family Systems: A Thematic Synthesis

**DOI:** 10.1111/cge.70102

**Published:** 2025-11-04

**Authors:** Paige Lindo, Tierney Tindall, Suzanne Buswell, Selina Lock, Sarah Gunn

**Affiliations:** ^1^ School of Psychology and Vision Sciences University of Leicester Leicester UK; ^2^ Learning and Library Services University of Leicester Leicester UK

**Keywords:** caregivers, experiences, family systems, Huntington's disease, psychosocial impact, relationships

## Abstract

Huntington's disease (HD) has prevalent, life‐altering consequences for affected individuals, relatives, familial caregivers and systemic functioning. However, the shared psychosocial impacts of HD across family systems are inadequately understood, and a synthesis of evidence regarding these experiences is currently lacking. This thematic synthesis provides an up‐to‐date integration of qualitative research describing psychological, social and relational difficulties experienced by HD families. A systematic search across PsycINFO, CINAHL, MEDLINE and Scopus identified nine qualitative studies. Four interconnected superordinate themes were developed, describing a disintegration of HD families from society, HD‐related emotional and psychological burdens, an interplay of extrinsic stressors and recalibration of the family system. These findings extend existing knowledge about systemic impacts of HD, highlighting diverse and pervasive psychological and social difficulties faced by families. The synthesis recommends the development of interventions and clinical understandings to appropriately support family systems around psychosocial and relationship dynamic challenges in the unique context of HD.

## Graphical abstract

The psychosocial impacts of Huntington's disease include disintegration with society, emotional and psychological burdens, an interplay of extrinsic stressors and recalibration of the family system. This thematic synthesis highlights the need for holistic, systemic support to address these pervasive challenges for all members of the affected family system.

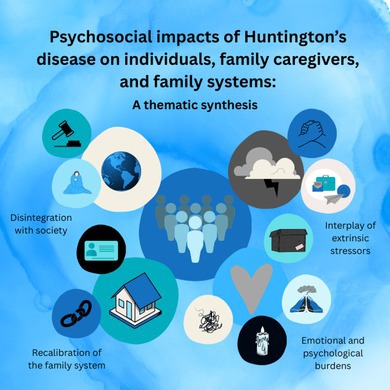

Huntington's disease (HD) has diverse and often dramatic impacts on affected families. People with HD (pwHD) have a typical life expectancy of 10–20 years from diagnosis [1], with onset usually around 30–50 years [2].

Diverse HD symptomology creates practical and emotional challenges for individuals and families. As HD progresses, pwHD typically experience motor difficulties such as chorea and rigidity, affecting speech, swallowing and breathing, creating health risks and limiting activities of daily living and leisure. HD‐related cognitive changes commonly affect executive function, processing speed, memory and learning [3]. Individuals may struggle to recognise these problems (anosognosia), creating risk and hampering communication within families and wider systems [4, 5]. Psychological and emotional difficulties are common, including low mood, irritability, anxiety, suicidal ideation and self‐harm [6]. Associated behavioural changes encompass social withdrawal and aggressive behaviour, creating barriers to participation and societal acceptance [3, 7, 8].

These physical, behavioural, cognitive and emotional difficulties create a challenging environment for pwHD, gene‐negative relatives and those at risk (including children). Recent large‐scale quantitative research demonstrates significant, understandable distress throughout HD families [9, 10], including around the 50% risk of inheritance for children [11, 12]. However, our current understanding of the psychosocial challenges and experiences of HD‐affected families is limited.

## 1 Psychological Impacts of HD

Effects of HD on pwHD and those at risk have been investigated to a degree across the lifespan. Influences of HD on psychological wellbeing can begin with decision‐making around diagnostic or predictive genetic testing, as this choice—and its outcome—is highly impactful for individuals and relatives [13]. Testing can empower pwHD by providing certainty, enabling future planning around life decisions and finances [14]. Contrastingly, testing may increase hopelessness or suicidal ideation for tested individuals [15, 16], and increased distress and hopelessness are also reported among partners of tested individuals post‐testing [17, 18].

Following testing, low mood appears more prevalent in earlier HD stages than later, potentially attributable to pwHD adjusting over time, and/or to impaired insight alongside progressing neurodegeneration [19]. Contrastingly, Dale and van Duijn [20] found no difference in anxiety levels in HD's early stages. Exuzides et al. [21] reported that pwHD have increased depression, anxiety and lower quality of life, and their partners have greater depression and anxiety compared to caregivers for people with other neurodegenerative diseases. Furthermore, relatives in HD families may experience emotional isolation due to prioritising the well‐being of pwHD, and are at risk of ‘anticipatory grief’ regarding themselves or future generations [22, 23].

People with later‐stage HD may experience increased obsessive‐compulsive symptoms, disorientation and apathy [9]. Importantly, this study found no significant differences between pwHD and relatives in the frequency of depression, suicidal ideation, hallucinations, aggression, irritability and anxiety. This emphasises that living with HD is highly distressing for the whole family. Overall, the familial psychological impacts of HD are evidently complex, and a holistic understanding of such impacts across family systems is required.

## 2 Prior Reviews

Limited literature reviews have explored HD's psychosocial consequences for affected families systemically. Domaradzki [24] reviewed the impacts of HD on family caregivers, identifying themes including family breakdown, worries for children and loss. Parekh et al. [25] extended these findings in their meta‐synthesis, unveiling emotional and practical difficulties, loss, risk concerns and relationship changes. Cooper et al. [26] explored young people's experiences of growing up with an HD‐affected parent, identifying themes around relationship difficulties, identity loss, lack of transparency and reclaiming lives, highlighting unique impacts on children and family dynamics.

Even fewer reviews have focused on experiences of pwHD themselves. In Mahmood et al. [27], themes were developed around avoidance, control, adaptation, coping and newfound respect for life. However, there was little exploration of systemic functioning and how psychosocial distress for pwHD and familial caregivers might hold reciprocity, despite the potential importance of these factors for mental wellbeing [28]. Prior reviews highlight a need for qualitative synthesis from an integrative, systemic perspective to better understand psychosocial experiences of HD‐affected families.

## 3 Objectives

Few reviews have explored the unique psychological, social and inter‐relational challenges of HD conjunctly, nor have any up‐to‐date reviews explored experiences of pwHD and familial caregivers collectively. Accordingly, we aim to develop novel and comprehensive insights into HD's psychosocial impacts for pwHD and familial caregivers, integrating their perspectives to examine impacts on family dynamics and systems collectively. This will illuminate HD‐specific difficulties requiring tailored intervention and support.

## 4 Materials and Methods

### 4.1 Search Strategy

Existing research was appraised in detail, following systematic search procedures. Consultation was undertaken with ‘HD Voice’ (a patient and public involvement group facilitated by the HD Association) to fine‐tune the research question and identify key areas for consideration.

The SPIDER framework [29] (Data S1: Supplementary File A) shaped the systematic search. A preliminary scoping search was undertaken using key terms ‘Huntington's’, ‘family’ and ‘impact’, with full search terms then developed with support from a specialist subject librarian (Data S1: Supplementary File B). Ethical approvals were not required.

### 4.2 Eligibility Criteria

Eligible studies were: peer‐reviewed empirical research exploring subjective experiences of HD (qualitative or mixed‐methods); published in English between 2004 and 2025 (to maximise temporal validity); focused on people who either had HD, were gene‐positive, were at risk, or were a relative providing psychological, emotional or physical support to a pwHD.

Studies were excluded which were: focused on alternative population(s); exploring factual, concrete elements of HD (e.g., its genetic underpinnings and biological symptoms); published in languages other than English and/or before 2004; non‐peer reviewed or preprints; conceptual or theoretical research; quantitative research; or secondary research (e.g., systematic reviews; meta‐analyses).

### 4.3 Study Selection

**FIGURE 1:**
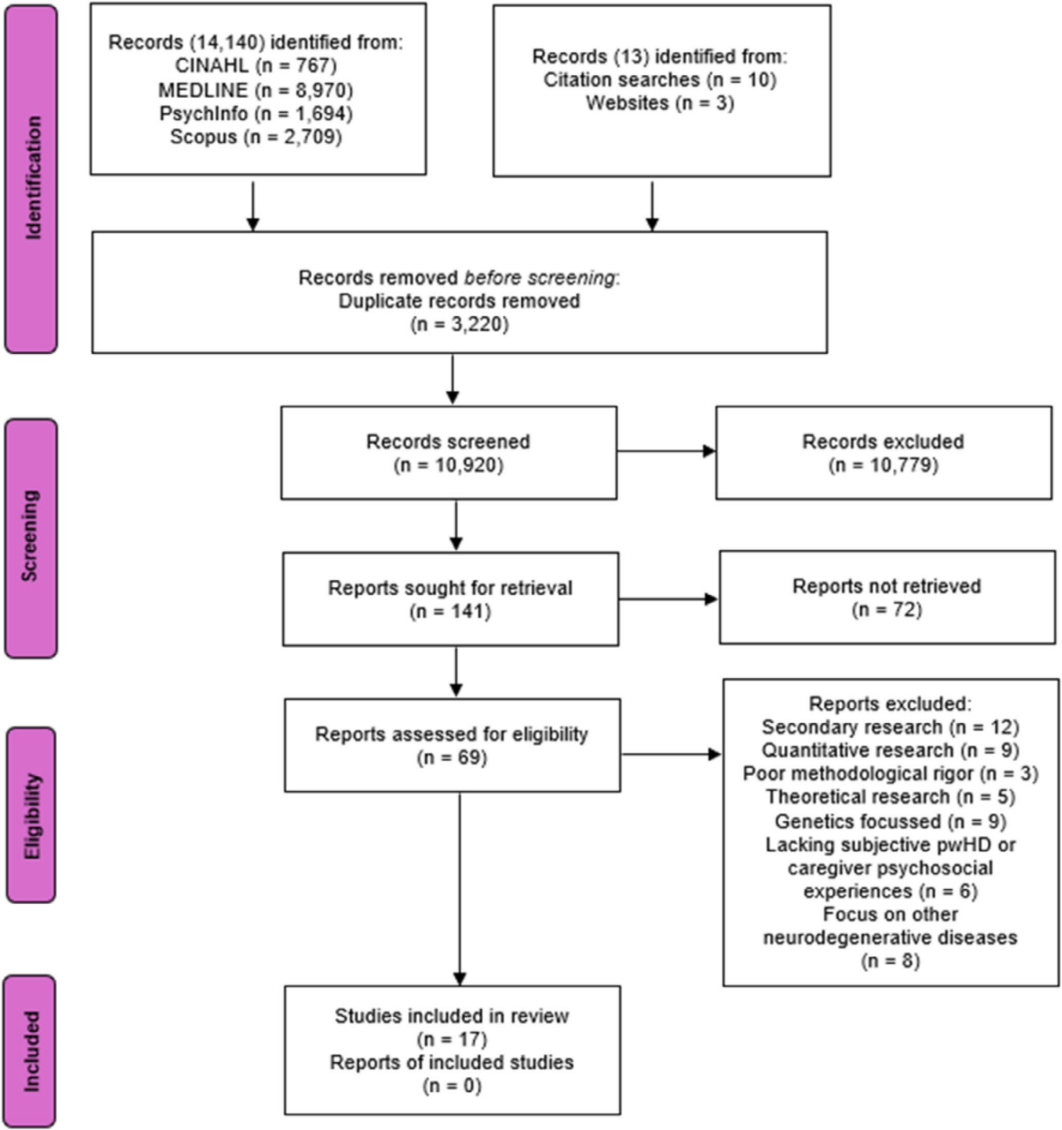
Study selection process. Summarises the study selection process using the PRISMA framework, mapping identification, screening, eligibility assessment and inclusion stages. PRISMA flowchart [30] depicting the screening stages.

A systematic search was undertaken by PL in May 2024 of four databases (PsycINFO, CINAHL, MEDLINE and Scopus), chosen due to their large scope and relevance in psychology and neuropsychiatry. An updated search was conducted by PL and SG independently in June 2025. Seventeen articles were identified (Figure 1).

### 4.4 Data Extraction and Appraisal

The Cochrane Data Extraction Tool was adapted to the review question and qualitative approach, and used to assess papers' eligibility for inclusion considering the aims, sample, data collection methods and analytic approach (Table 1). The JBI Tool was chosen to appraise full‐text articles due to its reliability and validity in assessing the quality and methodological rigour of qualitative and mixed‐methods studies [31]. Data extraction was completed by PL, consulting regularly with SG to support accuracy and consistency.

**TABLE 1:** Data extraction table comprising selected studies.

Study	Date	Location	Sample	Data collection	Analysis	Study themes/findings
Brewer et al.	2008	UK	12 parents/guardians of children with juvenile HD	Interviews	Interpretative phenomenological analysis	Dealing with something so different; a lack of understanding and isolation
Carney et al.	2025	Ireland	11 family caregivers	Interviews	Interpretative phenomenological analysis	The impact on caregivers; the impact of the genetic risk; accessing health care & support services
Daemen et al.	2025	The Netherlands	13 individuals with a HD‐affected parent	Focus groups	Thematic analysis	Experiences and impacts of growing up with a parent with HD; experiences with help/support; advice and support needs
Dawson et al.	2004	Australia	32 individuals (6 pwHD, 19 family caregivers, 7 healthcare professionals)	Interviews	Thematic analysis	Adjusting to the impact of illness; searching for essential information; gathering practical support; bolstering the spirit; choreographing individual care; future fears
Forrest Keenan et al.	2007	UK	33 young people (ages 9–28) from an HD‐affected family	Interviews	Grounded theory	Young people as carers; worry; coping; in need and at risk
Hubčíková et al.	2022	Slovak Republic	30 individuals recruited through the Slovak HD Society (9 pwHD, 5 at risk and 16 caregivers)	Questionnaires (open‐ended and multiple‐choice questions)	Grounded theory	Burdens for caregivers; relationship changes; financial difficulties
Kjoelaas et al.	2020	Norway	36 young people and adults who have grown up in an HD‐affected family	Interviews	Thematic analysis	Family functioning; emotions and reactions; social functioning; public/care services
Maxted et al.	2014	UK	7 parent/child dyads affected by HD	Interviews	Interpretative phenomenological analysis	HD's presence within the family; protection, knowledge and control; cyclical changes in identity/roles
Røthing et al.	2014	Norway	15 family caregivers of (recruited through Norwegian hospital departments and a lay organisation)	Interviews	Thematic analysis	Role/relationship transitions; caregivers adapting to emotions; vulnerable and fragmented family networks; age of onset with family life phases
Røthing et al.	2015	Norway	15 family caregivers	Interviews	Systematic text condensation	Regulating information; a skewed balance and increasing isolation; increasing limitations
Scerri	2015	Malta	8 family caregivers	Interviews	Grounded theory	The presence of HD; micro/macro conditions; resources/coping strategies
Smith et al.	2006	UK	12 parent caregivers of young people with juvenile HD	Interviews	Interpretative phenomenological analysis	Becoming aware that something is wrong; physical symptoms; speech/communication difficulties; behavioural problems; a slow, relentless process
Sparbel et al.	2008	US & Canada	32 teens (ages 14–18) in HD families	Focus groups	Content analysis	Watching and waiting; alone with others; family life is hard; having to be like an adult
Wieringa et al.	2022	UK	10 pre‐manifest HD adults	Interviews	Interpretative phenomenological analysis	Feeling time limited; stalling time; making the most of time
Williams et al.	2009	US & Canada	32 teens (ages 14–18) in HD families	Focus groups	Content analysis and narrative synthesis	Tasks/responsibilities; subjective burden; caregiving with personal risk; decisional responsibility
Williams et al.	2009	US & Canada	42 adult family caregivers (from 4 US and 2 Canadian centres between 2001 and 2005)	Focus groups	Grounded theory and thematic analysis	Life disintegration; relationship losses; risks for children; anticipating death
Williams et al.	2012	UK & US	108 UK and 112 US adult family caregivers	Surveys with narrative components	Deductive analysis	Isolation; role changes; concerns for children. UK participants had higher levels of sadness. US participants expressed higher worries for children and finances

### 4.5 Reflexivity

PL undertook data extraction and synthesis. SG independently coded a randomly selected 29% of papers (*n* = 5), enabling validation of themes via triangulation and comparison. This predominantly highlighted the convergence of developed themes between authors, and added nuance and depth to findings. PL/SG jointly reviewed all themes and underpinning data, discussing extensively to explore different understandings, interpretations and assumptions. PL maintained a reflexive log throughout, reflecting on potential influences of clinical, personal and professional experiences on interpretation and discussing with SG to ensure critical engagement with the data and minimise the impact of assumptions.

This review takes an interpretivist epistemological position, utilising thematic synthesis to generate new meanings and understandings of findings from the selected papers, with an inductive approach to develop themes. The interpretivist position was well‐suited to this review and methodology, aligning with the complexity, subjectivity and context‐specific psychosocial impacts of HD, whilst providing a framework for rich exploration of individual and familial HD‐related experiences, perspectives and meanings.

### 4.6 Analysis

Thomas and Harden's [32] thematic synthesis methodology was employed to flexibly explore individual experiences whilst maintaining depth of data in‐context, aiming to integrate findings and generate new insights, advancing understanding beyond individual study findings. Theme generation started with several re‐readings of texts for data familiarisation, followed by line‐by‐line manual coding (100% of papers by PL; 29% by SG), then collation of codes into initial themes. Themes were reviewed, and overarching themes were defined and named. The PRISMA checklist was applied to ensure transparency [30].

## 5 Results

### 5.1 Study Characteristics

The 17 papers were published between 2004 and 2025, all examining experiences of families impacted by HD. Overall, 560 pwHD, family caregivers, young people and people at risk of HD were included. Participants were assessed through interviews (*n* = 198), focus groups (*n* = 119), surveys (*n* = 220) and questionnaires (*n* = 30). Studies took place in the UK, Ireland, the Netherlands, USA, Canada, Australia, Slovak Republic, Malta and Norway. Analysis comprised grounded theory (*n* = 4); interpretative phenomenological analysis (*n* = 5); thematic analysis (*n* = 5); content analysis (*n* = 2); deductive analysis (*n* = 1); systematic text condensation (*n* = 1) and narrative synthesis (*n* = 1).

### 5.2 Meta‐Synthesis

Four superordinate themes were developed concerning impacts of HD on pwHD, caregivers and family systems: (1) disintegration with society, (2) emotional and psychological burdens, (3) an interplay of extrinsic stressors and (4) recalibration of the family system (Data S1: Supplementary Files D and E).

### 5.3 Disintegration With Society

This theme addressed social impacts of HD for affected families, encompassing both experienced separation from wider society and isolation within the family. Participants expressed feeling judged or misunderstood, and a general lack of HD knowledge from others. Many described actively withdrawing from peers, and feeling increasingly isolated from support networks. This theme comprised two subthemes: ‘stigma and social judgement’ and ‘isolation from others’.

#### 5.3.1 Stigma and Social Judgement

This subtheme captured how affected families carried the burden of ‘othering’ and others' judgements. Affected families reported perceiving those outside the family as different, and that others might see the pwHD differently due to their condition: ‘I see everybody else as different… whereas everybody else would see my mum as different but I don't’ [33, p. 125]. This connected to a perceived taboo around discussing HD with outsiders, fueling a disconnection from wider society: ‘You kind of do feel… the loneliest person in the room… it's like motor neuron's illegitimate brother… nobody wants to talk about’ [34, p. 6].

Participants noted stigma regarding behaviours and visible difficulties associated with HD (including family conflicts, aggression and symptom expression). One parent reflected on reactions to her daughter's motor movements, noticing that others ‘look as if to say oh, she's drunk’ [35, p. 9], with societal narratives inaccurately framing such behaviours as deviant or deliberate. These interactions influenced participants' perceptions of outsiders as judgemental, leading to exacerbated feelings of separation: ‘Everybody just stands there and looks at you… It's just like, “oh, what's she doing that for? If she were my child I'd give her such a crack”’ [36, p. 492]. Some participants internalised stigmatisation, experiencing shame and discomfort: ‘I was very hesitant to bring people home… it was embarrassing that other people should also see them fight’ [37, p. 135].

#### 5.3.2 Isolation From Others

In addition to disintegration from wider society, pwHD reported experiencing separation from family caregivers, feeling misunderstood and alone due to their different health and HD status: ‘I… heard from my husband that I am healthy and do not understand him’ [38, p. 8]. For caregivers, some expressed feeling unseen and unvalued due to cognitive and emotional changes in pwHD: ‘He… was mainly focused on himself and had no attention for me, which made me feel like I didn't matter’ [39, p. 95]. Additionally, caregivers acknowledged increased distance from relatives outside the household, due to their poorer HD awareness: ‘My uncle and his wife don't fully understand… and because of that, we've lost that part of the family’ [40, p. 332].

Participants described intentional and unintentional self‐isolation, alongside feeling pushed away by others. Many caregivers prioritised relationships with the pwHD, but this could create unintentional barriers to societal integration: ‘Me and my sister were best friends… I have not been able to establish friendships of my own’ [41, p. 702]. Some pwHD chose to keep peers at arm's length: ‘He… has made a decision not to see anybody… the burden is left with me to keep him company’ [42, 43], restricting social networks for the family. Finally, exclusion by others occurred when participants tried forming connections: ‘We had neighbours we tried to talk to… and there were friends of my dad's, but nobody reacted… People are very scared of meddling’ [37, p. 135]. Participants attributed this resistance to worries around becoming involved, and not knowing what to do or say, preventing HD families from accessing social support.

Conversely, one caregiver noted a consolidation of support within the HD community: ‘We have to unite to help each other; there is no other support for us’ [38, p. 8]. Support and understanding were only seen as available from those going through the same experience. Some HD family members also felt closer due to their struggles: ‘We were always close, but we, we, we feel it's us against the world, sometimes’ [44, p. 344]. This indicates potential for increased closeness and support in the context of HD for some, represented in a minority of studies.

### 5.4 Emotional and Psychological Burdens

This theme encapsulates the psychological implications of navigating life with HD. These are captured within four subthemes: ‘distress and overwhelm’ in the face of an incurable condition; ‘anxiety and fear’ around HD, risks, and the future; ‘grief, loss and mourning’ and ‘repression as a coping strategy’.

#### 5.4.1 Distress and Overwhelm

Distress and overwhelm were expressed by pwHD and relatives around HD's incurability. For some pwHD, distress manifested as low mood: ‘The diagnosis… had a tremendous impact on his life, and of course mine… he's been very depressed’ [45, p. 126]. There was also hopelessness at HD's slow, progressive deterioration: ‘The time period is so long and it's just this very slow walk down a very long road… each day is just slightly worse than the day before. And there is no end to the tunnel’ [42, 43].

From a systemic perspective, pwHD feared for impacts on their family. One person described their priority ‘that I wouldn't ruin my partner's life’ [38, p. 9]. Some family members noted HD's impacts on non‐gene‐carrying relatives: ‘Our daughter who stayed at home was like a chopping block for her HD affected mother. When something went wrong she was always the one to blame. She must have suffered a lot’ [41, p. 702]; ‘My wife has mainly mental symptoms […] I find the strain of trying to maintain some normality at home for my daughter overwhelming at times’ [46]. HD had clear impacts, not only on pwHD themselves and through their direct interactions with relatives, but through the anxieties of relatives for others (especially children).

Caregivers described low mood around caregiving demands: ‘I was actually diagnosed with depression from all the work, “cause I was sleep deprived from staying up with my dad, so…”’ [42, 43]. Participants expressed a range of distressing emotions, including ‘frustration’ and ‘hurt’, alongside reflections around having ‘suffered’ and feeling ‘sad’ [41, pp. 702–703], compounded into emotional overwhelm. Hopelessness was sometimes perceived from healthcare workers: ‘The neurologist didn't pay attention and immediately wrote us off as an incurable diagnosis’ [38, p. 8]; this compounded distress.

#### 5.4.2 Anxiety and Fear

Anxiety and fear were prevalent, particularly around anticipating symptom onset: ‘You forget things or names… it only takes a couple of things to happen… for you to start panicking and think “oh my god, is this it, is it starting?”’ [47, p. 379]. PwHD described anxiety and fear around the future, often around symptom progression, a ‘Sword of Damocles’ constantly in mind [44, p. 342]; ‘He can't face the fact that he saw his mother in the final stages and knows that along the track he'll be turning out like that’ [45, p. 128].

Both past experience and future expectations influenced participants' anxieties. Familial experiences of HD shaped individuals' fear of their own futures, and caused past and present trauma: ‘My father and sister were HD patients. I saw aggression, the desire to kill, they ruined my life’ [38, p. 9]. Participants also feared for future generations' risk of HD, influencing decisions around whether to have children: ‘From what I've seen my family members go through… I don't want to have kids. I don't want to risk giving it to somebody else’ [40, p. 331]. Participants with children experienced hypervigilance around their behaviours: ‘One of my kids spilled something, I thought, “Oh my God, no”’ [42, 43]. Caregivers also described their expectations for the future and the fears this incurred: ‘The hardest thought is that my beloved husband, who is very wise, rational, just, will become someone who cannot take care of himself and lose his identity’ [38, p. 9]; ‘I saw it in Mum, and, because I've seen it in Janet (…) we understand what's coming’ [44, p. 342]. Anxiety and fear were pervasive among the family system.

#### 5.4.3 Grief, Loss and Mourning

This subtheme reflected varying experiences of mourning, loss and grief among HD families. Participants described losses associated with activities and life before HD, including changing independence: ‘Can't drive, can't work, basically he couldn't do anything… suddenly he'd lost all’ [45, p. 126]. Caregivers expressed anticipatory grief, acknowledging that by the time death occurred, they would have already finished mourning due to the progressive losses sustained as the pwHD deteriorated: ‘I told somebody… “I'm done grieving… when my husband's gone, don't look for me to cry because that's what I've done already”’ [42, 43].

Participants who were caregivers in childhood reflected on lost innocence and grieved for a childhood not had: ‘They ruined my life, nobody will bring me back my youth’ [38, p. 9]. A parent expressed a related sentiment:

I don't think I'm a very good mother. I love my kids, but I think in their formative years, when they were in their teens when I should have been watching what they were doing and you know, I was not, my whole life was Julian.

Maxted et al. [44, pp. 345–346]

These quotes speak to systemically experienced losses, with grief expressed not only for the progressive losses associated with HD, but for time that cannot be recovered with other relatives.

#### 5.4.4 Repression as a Coping Strategy

Repression was a psychological coping strategy used by affected families. Caregivers noted that pwHD would try to ignore HD‐related experiences, which obstructed external support: ‘Given that she does not acknowledge the symptoms it is hard for people to help’ [46, p. 142]. Some caregivers reported similar emotions themselves, stating that they ‘used to not want to talk about it’ [37, p. 135], suggesting a familial collusive avoidance.

Some participants expressly recognised this protective mechanism to maintain wellbeing and defended its use: ‘What's wrong with blocking it out? Maybe that's what I've been doing for the last five years but it's worked quite well for me’ [44, p. 342]. Repression was perceived as mitigating psychological harm: ‘If you try and dwell on things too much… it makes you even worse off… I'd probably have a breakdown’ [35, p. 12]. It could thus be perceived positively, an active choice helping individuals cope with HD:

We decided to ignore the fear of the disease and live life as it is and as it will come. I don't want to be tested; I know what awaits me in the case of a positive test, and I don't want to wrap my life in the gloom of Huntington's disease.

Hubčíková et al. [38, p. 8]

Accordingly, repression was perceived as an adaptive strategy to maintain mental wellbeing against ongoing emotional turmoil.

### 5.5 An Interplay of Extrinsic Stressors

Two subthemes capture structural and systemic HD‐related challenges that intensified emotional distress: ‘unmet support needs’, including from healthcare services and ‘occupational and financial difficulties’ arising for pwHD and caregivers, impacting throughout the family.

#### 5.5.1 Unmet Support Needs

This subtheme encapsulated the absence of appropriate professional support: ‘There is so limited (a) knowledge, (b) expertise and (c) actual physical on the ground supports’ [34, p. 8]. Participants described encounters with services that lacked expertise and sensitivity to address HD‐specific challenges: ‘We would call the ambulance… they didn't understand… At times they would even suggest I was the one in need of psychiatric help’ [37, p. 136]. These interactions left participants feeling desperate and frustrated, causing ruptures with systems expected to provide compassionate support: ‘I just couldn't take it…. I lifted the table in front of him and I told the doctor: “at least try to understand me… you are making me lose my patience”’ [48, p. 24].

Problematic interactions saw participants withdrawing from support due to feeling unheard and misunderstood, even by specialist services: ‘I didn't get help from someone specialised in HD… they didn't understand me… I avoided help for a long time’ [39, p. 98]. Families consequently felt obliged to educate both themselves and medical staff: ‘There was no information… I searched for everything on the internet myself’; ‘I even had to actively explain to the general practitioner what the disease was and what it was causing’ [38, p. 8].

Participants also referenced lacking formal support from other non‐specialist sources, such as schools: ‘Nobody understood our situation. There was a lot of misunderstanding from others. It felt like nobody could help which made me feel sad’ [39]. Overall, this subtheme illustrates HD families feeling unsupported by many formal sources.

#### 5.5.2 Occupational and Financial Difficulties

Occupational difficulties were prominent for pwHD and caregivers. For pwHD, experiencing symptoms in the workplace engendered missed opportunities, loss, or withdrawal: ‘I haven't been able to find a job for a long time… with my involuntary movements… I had an accident almost everywhere at work’ [38, pp. 9–10]. Caregivers struggled to balance occupational or educational expectations with caregiving demands: ‘I missed most of my third year at school… I would skive off just to make sure she was all right’ [33, p. 122].

Occupational difficulties incurred financial struggles. PwHD expressed worries around the cost of living: ‘I receive benefits, which… are not even enough to cover the basic living costs’ [38, p. 10]. Caregivers echoed such concerns, reflecting on limited support from benefit systems: ‘My dad is under the age of sixty, we don't really qualify for Title 19 [health benefits]’ [42, 43]. Some participants described the emotional toll of trying to access financial support, expressing that ‘it's humiliating’ [34, p. 5]. Due to financial stresses and HD care costs, some participants maintained employment through necessity: ‘I had to work full‐time at retirement age… to pay for my husband's private caring facility’ [38, p. 9].

Behavioural changes influenced the spending habits of pwHD, amplifying financial concerns: ‘She's wanting to spend money… while it's in her bank she'll want to spend it’ [36, p. 492]. Caregivers described feeling increasingly alone with managing such difficulties, as the pwHD's ability to share the pragmatic or emotional load reduced as cognition declined [41].

### 5.6 Recalibration of the Family System

This theme encapsulates how HD disrupts and reshapes family dynamics, requiring pwHD and family caregivers to adjust relationally. Two subthemes were developed: ‘emotional and physical disconnect’, reflecting loss of intimacy and reciprocity and ‘adapting to new roles and identities’, where pwHD and caregivers redefined their place within the family and its dynamics.

#### 5.6.1 Emotional and Physical Disconnect

PwHD and caregivers described a disconnect within their families due to HD, with some deliberately physically distancing due to behavioural changes: ‘He was getting very aggressive… hitting the children… we couldn't cope with that’ [45, p. 127]. Increasing symptoms could also restrict physical intimacy, limiting these connections: ‘My wife is incapable sexually because of lack of concentration and she moves about’ [48, p. 23].

Previously supportive, emotionally intuitive and reciprocal relationships were perceived as deteriorating, with relatives feeling they no longer interacted meaningfully with the pwHD: ‘We haven't had a conversation in the last year and a half—like an actual conversation’ [40, p. 331]. Caregivers described creating emotional and physical separation from the pwHD, to cope: ‘I've kind of walled him off in so many ways… the physical, mental bond between a husband and wife, that's… gone’ [42, 43]. This disconnection was also expressed around changing personality associated with HD:

What hurts me most is that you lose the one you loved. A complete different person is sitting in the dining room today, another person than the one who was my husband years ago. That is sad for me. I am more like a caring person now, not his spouse and loved one as I was.

Røthing et al. [41, p. 702]

These increasing disconnections led some to contemplate separation: ‘I need to feel love from her but she is void of any emotions… I don't expect we will manage to remain a family much longer’ [46, p. 142]. This highlights the salience and impact of such disconnects within HD families; caregivers wished to support their loved one, but found the fading connection increasingly insupportable.

#### 5.6.2 Adapting to New Roles and Identities

Identities and roles within the family system shifted markedly as HD progressed, with family members taking on caring roles and losing prior positions as partner, sibling or child. Young caregivers voiced challenges and costs of stepping prematurely into adult roles: ‘I can't really be myself, I have to be [like] an adult… I want to be a brat… I want to be myself, and I can't’ [40, p. 332]. There were felt pressures to assume a parent's role to support family stability: ‘I tried to contribute a bit, like taking over my father's role’ [39, p. 96], highlighting parental role reversal and adultification for young caregivers of pwHD. Such role shifts were also evident in spousal relationships, with some recognising a transition of necessity from spousal identities to parent and child: ‘I love him dearly and I take care of him. But I've got this mother‐child relationship… I'm not in love with him anymore’ [42, 43].

Some participants' changing identity and roles thus appeared functional or deliberately chosen, to keep the family system working alongside impacts of HD. One participant described discomfort when this role was inhibited while accompanying their partner in a healthcare context: ‘The doctor did not let me into the office or tell me anything […] I was sorry because it is difficult for both my husband and me’ [38, p. 9]. This person considered themselves part of a dyad struggling together, but being excluded despite their caregiving role.

Some participants spoke of attempting to retain aspects of themselves, as their familial role was reshaped:

Having effectively given up my life to be a carer I need to spend at least six hours a day on a personal project to keep my own brain from seizing up. I also need to try keeping myself groomed and feeling it is worthwhile making the effort although no one is there to appreciate me anymore.

Williams et al. [46, p. 142]

One family member explained the battle to live well alongside HD: ‘We've got to live with it. But we don't have to let it dominate our lives’ [44, p. 342]. Another, who grew up with a parent with HD, described compensating for the impacts by redefining ‘normal’: ‘It might sound weird, but I got used to everything. What seemed normal kept changing for me’ [39, p. 97]. Accordingly, members of HD families experienced significant shifts in the roles they took within the family dynamic, and made varied attempts to retain or develop new aspects of themselves alongside HD.

## 6 Discussion

This review explored psychosocial impacts of HD on pwHD, relatives and familial caregivers and family systems. Four themes were identified, highlighting disintegration from wider society experienced by HD families, emotional and psychological burdens of HD for family systems, extrinsic stressors which heighten isolation and distress, and how family systems recalibrate in response via redefinition of roles and relationships between members. These findings extend existing knowledge by conceptualising HD‐related impacts in the context of familial dynamics, relationships and the wider system, rather than individual populations (largely pwHD and caregivers). Considering families holistically in the context of HD‐related mental wellbeing appears crucial for supporting all family members.

Our findings highlight established challenges for HD families, including impacts of stigma, isolation and psychological burdens (notably distress, grief and fear). These are known difficulties for pwHD and their caregivers separately [49, 50, 51]. Considered systemically, our findings provide key knowledge about impacts across the family system and its interactions with external entities. For example, Domaradzki [24] noted caregivers' experiences of social exclusion; our findings extend this by clarifying a sensed division between the HD community (who understand the struggles of HD families) and those ‘outside’ it (who do not). Understanding was generally found only among close family; other HD community members were the only trusted ‘external’ option, with formal sources such as healthcare and schools perceived as failing to provide meaningful and informed support.

Interactions with healthcare systems at times felt actively unhelpful for familial mental wellbeing. For example, families described understandable hopelessness against an incurable condition, which could be reinforced by clinical staff seemingly communicating that ‘nothing can be done’. Lack of clinician understanding also led to families feeling obliged to take responsibility for knowledge acquisition themselves, and for teaching clinicians about HD [38]. Such interactions led to frustration, desperation and avoidance of help‐seeking, even over long periods [39, 48].

The ‘distress and overwhelm’ subtheme highlighted emotional struggles of HD families. This tallies with quantitative evidence showing elevated depression and anxiety among pwHD and caregivers [21], and shared mental health difficulties across HD family members [9]. From our findings, it is demonstrably essential to consider mental wellbeing systemically, as mental health difficulties for pwHD impact throughout the family. Such difficulties do not only impact the pwHD and their relatives directly, but relatives without the gene expansion experience additive anxiety around the pwHD's impact on other relatives (especially children), contributing to heightened distress for all.

These findings argue clearly for interventions which address reciprocal dynamics within family systems. While the reciprocal impacts of mental wellbeing difficulties among HD families have yet to be explored explicitly, research from other contexts suggests that illness narratives around the strains of supporting a relative with mental wellbeing difficulties require families to engage in meaning‐making, adaptation, transformation and developing hope to effectively support the person and maintain their own mental wellbeing [52]. Exploring illness narratives and meaning‐making among HD family populations (separately and together) may accordingly have important implications for support.

Grief emerged as a key consideration in the context of HD, again under‐explored to date. The important role of anticipatory grief has been identified [22, 23], but ‘ambiguous loss’ also appears likely to be conceptually significant—that is, grief experienced at the psychological loss of a loved one who remains physically present [53]. Ambiguous loss affects caregivers of people with dementia [54] and prolonged disorders of consciousness [55], and was recently identified as relevant in caregivers' experiences specifically around apathy in pwHD [22]. This concept may be important in understanding mental wellbeing for HD family systems more broadly, given the progressive changes in personality, behaviour, emotion and cognition which can leave relatives feeling that the pwHD has changed beyond recognition.

Grief was additionally expressed for lost time and relationships with other relatives, notably parents regretting opportunities for time with young children which were lost due to caregiving responsibilities [44]. This consolidates the importance of loss and grief in understanding systemic impacts of HD, and the necessity of considering grief in multiple forms including around the physical death of the pwHD, progressive losses prior, and other less tangible but also important losses within the family system.

Repression was identified as a key strategy for coping with HD and its impacts. This is little explored, although Mahmood et al. [27] noted avoidance among pwHD. Our synthesis reports how caregivers also express emotional disconnection for self‐protection, potentially interacting with repression from the pwHD in a reciprocal relationship [41], and even creating familial collusion in avoiding acknowledging HD. Such avoidance was expressly defended by some as an active choice to live well alongside HD, mitigating its impacts on quality of life.

Our findings around ‘occupational and financial difficulties’ corroborate past findings around caregivers' financial strain [38] and employment disruptions among pwHD [56]. While these difficulties are well known among the HD community, our findings highlight important emotional components to these losses—notably the painful changing dynamic between pwHD and caregiver, in which the caregiver shoulders the emotional and pragmatic burdens of familial financial and occupational changes, with the pwHD no longer able to share these decisions as they once did.

‘Recalibration of the family system’ builds on prior reviews identifying familial shifts in roles and relationships around HD [25, 26], integrating perspectives across HD families. Grounded in Family Systems Theory [57], we highlight how HD disrupts systemic functioning, with one member's experiences reshaping others' roles and emotional experiences. For example, the changing ability of pwHD to express appreciation and affection resulted in caregivers describing grief, isolation and lack of shared load in difficult times. This included spouses losing their long‐term partner as they moved into a ‘caregiver and cared‐for’ dynamic, to children becoming caregivers to pwHD and/or support to non‐HD parents.

Also important was tension between caregivers seeing themselves as part of a dyad, but being excluded in healthcare contexts (where the pwHD was supported individually). A tension was further evident between multiple ‘pulls’, with caregivers wanting to support their loved one, struggling with caregiving demands and the changing pwHD, and striving to retain aspects of themselves and live well alongside HD despite the challenges.

### 6.1 Critique of Studies

Four studies utilised group interviews, potentially impacting what individuals felt able to disclose. Most studies employed small sample sizes, providing minimal demographic and social information. From the data provided, there appeared to be a bias towards female spousal caregiver participants, potentially limiting the relevance of findings to other populations. Further, several studies sampled via HD organisations, presumably capturing the voices of a specific subset of HD‐affected individuals who engage with third sector and support organisations, which may not entirely represent wider HD populations.

Considering reflexivity, only study clearly acknowledged the authors' position and potential impacts upon the research. While this raises questions about transparency, it reflects historical publishing norms that may not have prioritised reflexivity, and all studies nonetheless displayed strong methodological rigour and adherence to high ethical standards.

### 6.2 Strengths and Limitations

Studies included were high quality, methodologically rigorous and drawn from varied databases and established journals. However, cross‐cultural validity was limited due to the exclusion of non‐English studies, restricting the inclusion of alternative cultural experiences. Nevertheless, included studies spanned globally from culturally diverse areas, bolstering cross‐cultural validity. Overall, 560 responses were analysed, providing a larger, more representative exploration of experiences contrasted with individual studies.

This review was inclusive across HD families and HD stages, providing broad perspectives on HD's impacts across the condition trajectory; however, experiences among specific subgroups and stages may vary. Moreover, most studies highlighted caregivers' perceptions of pwHDs' experiences, which potentially differ from pwHD's actual experiences.

### 6.3 Future Research

Future investigations could examine the impacts of HD on pwHD specifically, especially in systemic contexts, as caregiver perspectives dominated the identified studies. Exploring HD's psychosocial effects in more homogenous specific populations (e.g., juvenile‐onset HD or specific HD stages, for pwHD and caregivers) will support the development of more nuanced understandings. Little research has explored the impacts of culture; thus, future studies should also investigate cultural differences in HD‐related psychosocial distress.

Moreover, further investigation of ambiguous grief and impacts of illness narratives on relationships and mental wellbeing among HD families is strongly indicated. Additionally, research into factors which predict and support increased closeness for HD family members would be valuable, whereas some relatives described HD as having brought them together, more participants reported increased tensions and difficulties, and understanding these differences may hold theoretical and clinical value. There has further been inadequate attention to influences of past experiences of HD and future expectations, among pwHD and caregivers, but this played a key role in wellbeing for both.

### 6.4 Clinical Implications

Improving understandings among healthcare workers of the unique experiences associated with HD appears crucial, as the burden of explaining HD to non‐expert healthcare workers appears to add struggles to an already difficult situation for families. This may include developing HD educational resources for non‐specialist practitioners.

Given HD's systemic impacts reported here, HD‐specific interventions are indicated to address psychosocial distress, and provide support in the context of changing family dynamics and roles. Acceptance and Commitment Therapy, with its focus on living well alongside difficulties, may be clinically valuable [58]. Interventions and support should be provided holistically to pwHD, caregivers and relatives, considering the entire system. Where providing formal systemic support is unfeasible, exploring HD's impacts across wider systems should be integral to individual therapeutic work.

Clinical messaging around HD to families should be considered. Although communicating HD's (current) incurability is unarguably necessary for families to adequately understand the situation, families feeling ‘written off’ [38] is clearly harmful, neglects potential for symptom remediation, and potentially makes help‐seeking less likely as described above. By addressing psychosocial difficulties among HD‐affected families, individuals' quality of life could likely be enhanced, so healthcare staff might emphasise that while HD is currently incurable, management and mitigation of some difficulties can be achieved with appropriate strategies and support.

## 7 Conclusion

This synthesis captured the psychosocial impacts of HD across HD families, integrating diverse family experiences. Results extended past findings, recognising that HD caused a pervasive strain upon family systems in myriad forms. Families struggled with a lack of external support (formal and informal), financial and occupational challenges and feeling isolated from wider communities, but also grappled with increasing internal tensions and emotional stressors as HD progressed. Family members experienced the reshaping of their roles and identities, sought coping strategies and attempted to regain aspects of themselves in the context of these changing systemic contexts.

The findings highlight a need to further understand the perspectives of pwHD, family caregivers, young people, and those at risk, pointing to the crucial importance of understanding and treating HD wellbeing in the context of family dynamics. This is vital to develop meaningful and effective systemic interventions that are inclusive of all family members, to improve the quality of life for all affected by HD.

## Conflicts of Interest

The authors declare no conflicts of interest.

## Supporting information


**Data S1:** cge70102‐sup‐0001‐supinfo.docx.

## Data Availability

Data sharing not applicable to this article as no datasets were generated or analysed during the current study.

## References

[1] A. Sturrock and B. R. Leavitt , “The Clinical and Genetic Features of Huntington Disease,” Journal of Geriatric Psychiatry and Neurology 23, no. 4 (2010): 243–259. 20923757 10.1177/0891988710383573

[2] M. J. Novak and S. J. Tabrizi , “Huntington's Disease,” British Medical Journal 340 (2010): c3109. 20591965 10.1136/bmj.c3109

[3] R. A. Roos , “Huntington's Disease: A Clinical Review,” Orphanet Journal of Rare Diseases 5 (2010): 1–8. 21171977 10.1186/1750-1172-5-40 PMC3022767

[4] C. M. Jona , I. Labuschagne , E. C. Mercieca , et al., “Families Affected by Huntington's Disease Report Difficulties in Communication, Emotional Involvement, and Problem Solving,” Journal of Huntington's Disease 6, no. 3 (2017): 169–177. 10.3233/JHD-170250 PMC5682576 28968240

[5] E. J. Sitek , J. C. Thompson , D. Craufurd , and J. S. Snowden , “Unawareness of Deficits in Huntington's Disease,” Journal of Huntington's Disease 3, no. 2 (2014): 125–135. 10.3233/JHD-140109 25062855

[6] H. Bilal , N. Warren , P. Dahanayake , W. Kelso , S. Farrand , and J. C. Stout , “The Lived Experiences of Depression in Huntington's Disease: A Qualitative Study,” Journal of Huntington's Disease 11, no. 3 (2022): 321–335. 10.3233/JHD-220537 35570497

[7] S. Jauhar and S. Ritchie , “Psychiatric and Behavioural Manifestations of Huntington's Disease,” Advances in Psychiatric Treatment 16, no. 3 (2010): 168–175.

[8] J. S. Paulsen , R. E. Ready , J. M. Hamilton , M. S. Mega , and J. L. Cummings , “Neuropsychiatric Aspects of Huntington's Disease,” Journal of Neurology, Neurosurgery & Psychiatry 71, no. 3 (2001): 310–314. 11511702 10.1136/jnnp.71.3.310 PMC1737562

[9] S. Gunn , M. Dale , N. Ovaska‐Stafford , and J. Maltby , “Mental Health Symptoms Among Those Affected by Huntington's Disease: A Cross‐Sectional Study,” Brain and Behavior: A Cognitive Neuroscience Perspective 13 (2023): e2954. 10.1002/brb3.2954 PMC10097147 36880126

[10] J. Maltby , N. Ovaska‐Stafford , and S. Gunn , “The Structure of Mental Health Symptoms in Huntington's Disease: Comparisons With Healthy Populations,” Journal of Clinical and Experimental Neuropsychology 43, no. 7 (2021): 737–752. 34906020 10.1080/13803395.2021.2002824

[11] H. Etchegary , “Coping With Genetic Risk: Living With Huntington Disease (HD),” Current Psychology 28 (2009): 284–301.

[12] N. Fahy , C. Rice , N. Lahiri , R. Desai , and J. Stott , “Genetic Risk for Huntington Disease and Reproductive Decision‐Making: A Systematic Review,” Clinical Genetics 104, no. 2 (2023): 147–162. 37095632 10.1111/cge.14345

[13] D. Craufurd , R. MacLeod , M. Frontali , et al., “Diagnostic Genetic Testing for Huntington's Disease,” Practical Neurology 15, no. 1 (2015): 80–84. 25169240 10.1136/practneurol-2013-000790

[14] B. Meiser and S. Dunn , “Psychological Impact of Genetic Testing for Huntington's Disease: An Update of the Literature,” Journal of Neurology, Neurosurgery & Psychiatry 69, no. 5 (2000): 574–578. 11032605 10.1136/jnnp.69.5.574 PMC1763433

[15] C. Mastromauro , R. H. Myers , B. Berkman , J. M. Opitz , and J. F. Reynolds , “Attitudes Toward Presymptomatic Testing in Huntington Disease,” American Journal of Medical Genetics 26, no. 2 (1987): 271–282. 2949611 10.1002/ajmg.1320260205

[16] A. Tibben , H. J. Duivenvoorden , M. F. Niermeijer , M. Vegter‐van der Vlis , R. A. Roos , and F. Verhage , “Psychological Effects of Presymptomatic DNA Testing for Huntington's Disease in the Dutch Program,” Psychosomatic Medicine 56, no. 6 (1994): 526–532. 7871108 10.1097/00006842-199411000-00008

[17] K. A. Quaid and M. K. Wesson , “Exploration of the Effects of Predictive Testing for Huntington Disease on Intimate Relationships,” American Journal of Medical Genetics 57, no. 1 (1995): 46–51. 7645597 10.1002/ajmg.1320570111

[18] A. Tibben , R. Timman , E. C. Bannink , and H. J. Duivenvoorden , “Three‐Year Follow‐Up After Presymptomatic Testing for Huntington's Disease in Tested Individuals and Partners,” Health Psychology 16, no. 1 (1997): 20–35. 9028813 10.1037//0278-6133.16.1.20

[19] J. S. Paulsen , C. Nehl , K. F. Hoth , et al., “Depression and Stages of Huntington's Disease,” Journal of Neuropsychiatry and Clinical Neurosciences 17, no. 4 (2005): 496–502. 16387989 10.1176/jnp.17.4.496

[20] M. Dale and E. van Duijn , “Anxiety in Huntington's Disease,” Journal of Neuropsychiatry and Clinical Neurosciences 27, no. 4 (2015): 45, 10.1176/appi.neuropsych.14100265. 25803201

[21] A. Exuzides , J. E. Matos , A. M. Patel , A. A. Martin , B. Ricker , and D. Bega , “Understanding the Burdens Associated With Huntington's Disease in Manifest Patients and Care Partners—Comparing to Parkinson's Disease and the General Population,” Brain Sciences 12, no. 2 (2022): 161. 35203927 10.3390/brainsci12020161 PMC8869871

[22] S. L. Mason , R. A. Barker , K. Andresen , F. Gracey , and C. Ford , “The Meaning of Apathy in Huntington's Disease: A Qualitative Study of Caregiver Perspectives,” Neuropsychological Rehabilitation 35, no. 5 (2024): 1004–1033. 39102382 10.1080/09602011.2024.2384519

[23] A. Tibben , M. V. V. D. Vlis , M. I. Skraastad , et al., “DNA‐Testing for Huntington's Disease in the Netherlands: A Retrospective Study on Psychosocial Effects,” American Journal of Medical Genetics 44, no. 1 (1992): 94–99. 1387764 10.1002/ajmg.1320440122

[24] J. Domaradzki , “The Impact of Huntington Disease on Family Carers: A Literature Overview,” Psychiatria Polska 49, no. 5 (2015): 931–944. 26688844 10.12740/PP/34496

[25] R. Parekh , R. T. Praetorius , and A. Nordberg , “Carers' Experiences in Families Impacted by Huntington's Disease: A Qualitative Interpretive Meta‐Synthesis,” British Journal of Social Work 48, no. 3 (2018): 675–692.

[26] H. Cooper , J. Simpson , M. Dale , and F. J. Eccles , “Experiences of Young People Growing Up in a Family With Huntington's Disease: A Meta‐Ethnography of Qualitative Research,” Journal of Genetic Counseling 34, no. 1 (2025): e1886. 38469914 10.1002/jgc4.1886 PMC11726609

[27] S. Mahmood , S. Law , and Y. Bombard , “‘I Have to Start Learning How to Live With Becoming Sick’: A Scoping Review of the Lived Experiences of People With Huntington's Disease,” Clinical Genetics 101, no. 1 (2022): 3–19. 34216010 10.1111/cge.14024

[28] M. E. Kerr and M. Bowen , One Family's Story: A Primer on Bowen Theory (Bowen Center for the Study of the Family, Georgetown Family Center, 2003).

[29] A. Cooke , D. Smith , and A. Booth , “Beyond PICO: The SPIDER Tool for Qualitative Evidence Synthesis,” Qualitative Health Research 22, no. 10 (2012): 1435–1443. 22829486 10.1177/1049732312452938

[30] M. J. Page , J. E. McKenzie , P. M. Bossuyt , et al., “The PRISMA 2020 Statement: An Updated Guideline for Reporting Systematic Reviews,” International Journal of Surgery 88 (2021): 105906. 33789826 10.1016/j.ijsu.2021.105906

[31] K. Hannes , C. Lockwood , and A. Pearson , “A Comparative Analysis of Three Online Appraisal Instruments' Ability to Assess Validity in Qualitative Research,” Qualitative Health Research 20, no. 12 (2010): 1736–1743. 20671302 10.1177/1049732310378656

[32] J. Thomas and A. Harden , “Methods for The Thematic Synthesis of Qualitative Research in Systematic Reviews,” BMC Medical Research Methodology 8 (2008): 45, 10.1186/1471-2288-8-45. 18616818 PMC2478656

[33] * K. Forrest Keenan , Z. Miedzybrodzka , E. Van Teijlingen , L. McKee , and S. A. Simpson , “Young People's Experiences of Growing Up in a Family Affected by Huntington's Disease,” Clinical Genetics 71, no. 2 (2007): 120–129. 17250660 10.1111/j.1399-0004.2006.00702.x

[34] * S. Carney , N. Pender , and E. Rogers , “Huntington's Disease Caregivers: A Qualitative Exploration of Caregivers Experience,” Journal of Health Psychology (2025): 13591053251328934, 10.1177/13591053251328934. PMC12881155 40243099

[35] * H. M. Brewer , V. Eatough , J. A. Smith , C. A. Stanley , N. W. Glendinning , and O. W. Quarrell , “The Impact of Juvenile Huntington's Disease on the Family: The Case of a Rare Childhood Condition,” Journal of Health Psychology 13, no. 1 (2008): 5–16. 18086713 10.1177/1359105307084307

[36] * J. A. Smith , H. M. Brewer , V. Eatough , C. A. Stanley , N. W. Glendinning , and O. W. J. Quarrell , “The Personal Experience of Juvenile Huntington's Disease: An Interpretative Phenomenological Analysis of Parents' Accounts of the Primary Features of a Rare Genetic Condition,” Clinical Genetics 69, no. 6 (2006): 486–496. 16712700 10.1111/j.1399-0004.2006.00624.x

[37] * S. Kjoelaas , K. H. Tillerås , and K. B. Feragen , “The Ripple Effect: A Qualitative Overview of Challenges When Growing Up in Families Affected by Huntington's Disease,” Journal of Huntington's Disease 9, no. 2 (2020): 129–141. 10.3233/JHD-190377 PMC7369039 32065801

[38] * K. Hubčíková , T. Rakús , A. Mühlbäck , et al., “Psychosocial Impact of Huntington's Disease and Incentives to Improve Care for Affected Families in the Underserved Region of the Slovak Republic,” Journal of Personalized Medicine 12, no. 12 (2022): 1941. 36556162 10.3390/jpm12121941 PMC9783383

[39] * M. M. Daemen , A. A. Duits , L. B. van der Meer , et al., “Through Their Eyes: A Retrospective Mixed‐Methods Study on the Experiences and Support Needs of Children Growing Up With a Parent With Huntington's Disease,” Journal of Huntington's Disease 14, no. 1 (2025): 93–102. 10.1177/18796397241304333 PMC12231935 39973392

[40] * K. J. Sparbel , M. Driessnack , J. K. Williams , et al., “Experiences of Teens Living in the Shadow of Huntington Disease,” Journal of Genetic Counseling 17 (2008): 327–335. 18347962 10.1007/s10897-008-9151-6 PMC2811873

[41] * M. Røthing , K. Malterud , and J. C. Frich , “Caregiver Roles in Families Affected by Huntington's Disease: A Qualitative Interview Study,” Scandinavian Journal of Caring Sciences 28, no. 4 (2014): 700–705. 24237139 10.1111/scs.12098

[42] * J. K. Williams , L. Ayres , J. Specht , K. Sparbel , and M. L. Klimek , “Caregiving by Teens for Family Members With Huntington Disease,” Journal of Family Nursing 15, no. 3 (2009): 273–294. 19465560 10.1177/1074840709337126 PMC4882923

[43] * J. K. Williams , H. Skirton , J. S. Paulsen , et al., “The Emotional Experiences of Family Carers in Huntington Disease,” Journal of Advanced Nursing 65, no. 4 (2009): 789–798. 19228233 10.1111/j.1365-2648.2008.04946.x PMC3807601

[44] * C. Maxted , J. Simpson , and S. Weatherhead , “An Exploration of the Experience of Huntington's Disease in Family Dyads: An Interpretative Phenomenological Analysis,” Journal of Genetic Counseling 23 (2014): 339–349. 24214466 10.1007/s10897-013-9666-3

[45] * S. Dawson , L. J. Kristjanson , C. M. Toye , and P. Flett , “Living With Huntington's Disease: Need for Supportive Care,” Nursing & Health Sciences 6, no. 2 (2004): 123–130. 15130098 10.1111/j.1442-2018.2004.00183.x

[46] * J. K. Williams , H. Skirton , J. J. Barnette , and J. S. Paulsen , “Family Carer Personal Concerns in Huntington Disease,” Journal of Advanced Nursing 68, no. 1 (2012): 137–146. 21668480 10.1111/j.1365-2648.2011.05727.x PMC3175316

[47] * G. Wieringa , M. Dale , and F. J. Eccles , “The Experience of a Sample of Individuals in the United Kingdom Living in the Pre‐Manifest Stage of Huntington's Disease: An Interpretative Phenomenological Analysis,” Journal of Genetic Counseling 31, no. 2 (2022): 375–387. 34374465 10.1002/jgc4.1497

[48] * R. Scerri , “Living With Advanced‐Stage Huntington's Disease: An Exploration of the Experiences of Maltese Family Caregivers,” British Journal of Neuroscience Nursing 11, no. 1 (2015): 20–27.

[49] A. Aubeeluck , “Caring for the Carers: Quality of Life in Huntington's Disease,” British Journal of Nursing 14, no. 8 (2005): 452–454. 15924027 10.12968/bjon.2005.14.8.17929

[50] C. M. Mand , L. Gillam , R. E. Duncan , and M. B. Delatycki , “‘I'm Scared of Being Like Mum’: The Experience of Adolescents Living in Families With Huntington Disease,” Journal of Huntington's Disease 4, no. 3 (2015): 209–217. 10.3233/JHD-150148 26443924

[51] R. A. Paoli , A. Botturi , A. Ciammola , et al., “Neuropsychiatric Burden in Huntington's Disease,” Brain Sciences 7, no. 6 (2017): 67. 28621715 10.3390/brainsci7060067 PMC5483640

[52] A. Cole , J. A. Pooley , and L. Whitehead , “Family Members' Experiences With Depression Through the Lens of Frank's Illness Narratives,” Collegian 31, no. 6 (2024): 392–403.

[53] P. Boss , Ambiguous Loss: Learning to Live With Unresolved Grief (Harvard University Press, 1999).

[54] A. Lindauer and T. A. Harvath , “Pre‐Death Grief in the Context of Dementia Caregiving: A Concept Analysis,” Journal of Advanced Nursing 70, no. 10 (2014): 2196–2207. 24702153 10.1111/jan.12411

[55] Y. Zaksh , E. Yehene , M. Elyashiv , and A. Altman , “Partially Dead, Partially Separated: Establishing the Mechanism Between Ambiguous Loss and Grief Reaction Among Caregivers of Patients With Prolonged Disorders of Consciousness,” Clinical Rehabilitation 33, no. 2 (2019): 345–356. 30255716 10.1177/0269215518802339

[56] D. I. Helder , A. A. Kaptein , G. M. J. van Kempen , J. C. van Houwelingen , and R. A. C. Roos , “Impact of Huntington's Disease on Quality of Life,” Movement Disorders 16, no. 2 (2001): 325–330. 11295789 10.1002/mds.1056

[57] N. Luhmann , Social Systems (Stanford University Press, 1995).

[58] S. Buswell , P. Lindo , and S. Gunn , “Acceptance and Commitment Therapy for People Affected by Huntington's Disease,” in Palgrave Encyclopedia of Disability (Springer, 2025), 1–12, https://link.springer.com/rwe/10.1007/978‐3‐031‐40858‐8_419‐1.

