# Supplementary material for: Psychosocial Impacts of Huntington's Disease on Individuals, Relatives and Family Systems: A Thematic Synthesis
Source: Clin Genet. 2025 Nov 4;109(3):403–15. doi: 10.1111/cge.70102 (PMC12881214; doi:10.1111/cge.70102)
Supplement: Supplementary file 1 — Data S1: cge70102‐sup‐0001‐supinfo.docx. [file CGE-109-403-s001.docx]

**Supplementary File A:** SPIDER Framework Shaping the Search Strategy

| SPIDER Framework | |
| --- | --- |
| S – Sample | Individuals with a diagnosis of HD, and/or family members providing physical or emotional support and care in any capacity (including those who may self-identify as a caregiver) |
| PI – Phenomenon of Interest | Studies exploring the impact of HD on an individual, family member/caregiver, and family dynamics level |
| D – Design | Research utilising questionnaires and/or surveys, interviews, and focus groups |
| E – Evaluation | Studies focused on subjective experiences with HD (including quality of life; interpersonal relationships; psychosocial changes) |
| R – Research Type | Qualitative or mixed methods |
|  |  |

**Supplementary File B:** Table of Identified Search Terms for the Systematic Search

| **Search terms** | **AND** | **AND** |
| --- | --- | --- |
| huntington* | impact OR | famil* OR |
|  | effect* OR | care* OR |
|  | consequence* OR | partner* OR |
|  | challenge* OR | spouse* OR |
|  | difficult* OR | child* OR |
|  | experience* OR | young pe* OR |
|  | distress* | offspring OR |
|  | psycho* OR | patient* OR |
|  | social* OR | client* OR |
|  | relation* OR | resident* OR |
|  | dynamic* | parent* |
| *Note.* * = truncation/letter substitution | | |

**Supplementary File C:** Quality Assessment Table for Eligible Studies

|  | **1** | **2** | **3** | **4** | **5** | **6** | **7** | **8** | **9** | **10** | **11** | **12** | **13** | **14** | **15** | **16** | **17** |
| --- | --- | --- | --- | --- | --- | --- | --- | --- | --- | --- | --- | --- | --- | --- | --- | --- | --- |
| **Is there congruity between the stated philosophical perspective and the research methodology?** | N | Y | Y | Y | Y | N | N | N | N | N | Y | N | N | Y | U | U | N |
| **Is there congruity between the research methodology and the research question or objectives?** | Y | Y | Y | Y | Y | Y | Y | Y | Y | Y | Y | Y | Y | Y | Y | Y | Y |
| **Is there congruity between the research methodology and the methods used to collect data?** | Y | Y | Y | Y | Y | Y | Y | Y | Y | Y | Y | Y | Y | Y | Y | Y | Y |
| **Is there congruity between the research methodology and the representation and analysis of data?** | Y | Y | Y | Y | Y | Y | Y | Y | Y | Y | Y | Y | Y | Y | Y | Y | Y |
| **Is there congruity between the research methodology and the interpretation of results?** | Y | Y | Y | Y | Y | Y | Y | Y | Y | Y | Y | Y | Y | Y | Y | Y | Y |
| **Is there a statement locating the researcher culturally or theoretically?** | N | Y | U | N | N | N | Y | Y | N | Y | N | N | N | N | N | N | N |
| **Is the influence of the researcher on the research, and vice-versa, addressed?** | Y | Y | Y | Y | Y | Y | Y | Y | Y | Y | Y | Y | Y | Y | Y | Y | N |
| **Are participants, and their voices, adequately represented?** | Y | Y | Y | Y | Y | Y | Y | Y | Y | Y | Y | Y | Y | Y | Y | Y | Y |
| **Is the research ethical according to current criteria or, for recent studies, and is there evidence of ethical approval by an appropriate body?** | Y | Y | Y | Y | Y | Y | Y | Y | Y | Y | Y | Y | Y | Y | Y | Y | Y |
| **Do the conclusions drawn in the research report flow from the analysis, or interpretation, of the data?** | Y | Y | Y | Y | Y | Y | Y | Y | Y | Y | Y | Y | Y | Y | Y | Y | Y |
| **Quality assessment score** | 8/10 High | 10/10 High | 9/10 High | 9/10  High | 9/10  High | 8/10 High | 9/10  High | 9/10  High | 8/10  High | 9/10 High | 9/10  High | 8/10 High | 8/10 High | 9/10 High | 8/10 High | 8/10  High | 7/10 Medium |

*Note.* Y = Yes; N = No; U = Unclear. 1. Brewer et al., 2008; 2. Carney et al., 2025; 3. Daemen et al., 2025; 4. Dawson et al., 2004; 5. Forrest Keenan et al., 2007; 6. Hubčíková et al., 2022; 7. Kjoelaas et al., 2020; 8. Maxted et al., 2014; 9. Røthing et al., 2014; 10. Røthing et al., 2015; 11. Scerri, 2015; 12. Smith et al., 2006; 13. Sparbel et al., 2008; 14. Wieringa et al., 2021; 15. Williams et al., 2009; 16. Williams et al., 2009; 17. Williams et al., 2012

Røthing M. Malterud K. Frich J. C. (2015). Balancing Needs as a Family Caregiver in Huntington's Disease: A Qualitative Interview Study. *Health & Social Care in the Community,* *23*(5), 569-576

**Supplementary File D:** Themes and Subthemes

| **Superordinate themes** | **Subthemes** |
| --- | --- |
| Disintegration with society | Stigma and social judgement |
|  | Isolation from others |
| Emotional and psychological burdens | Distress and overwhelm |
|  | Grief, loss and mourning |
|  | Repression |
|  | Anxiety and fear |
| An interplay of extrinsic stressors | Unmet support needs |
|  | Occupational and financial difficulties |
| Recalibration of the family system | Emotional and physical disconnect |
|  | Adapting to new roles and identities |

**Supplementary File E:** Sample Extract of the Theme Development Process Stemming from Participant Quotes

| **Superordinate themes** | **Subthemes** | **Code** | **Example quote** |
| --- | --- | --- | --- |
| Disintegration with society | Stigma and social judgement | HD is seen as a stigmatised condition that no one speaks about.  Negative judgement from others in the face of uncontrollable symptoms. | “You kind of do feel, you know, the loneliest person in the room… it’s like motor neurons illegitimate brother or illegitimate sister, nobody wants to talk about” (Carney et al., 2025, p.6).   “Everybody just stands there and looks at you… It’s just like, ‘oh, what’s she doing that for? If she were my child I’d give her such a crack’” (Smith et al., 2006, p.492). |
|  | Isolation from others | Loss of emotional and attentional reciprocity.    A lack of understanding creates family distance. | “He [parent with HD] was mainly focused on himself and had no attention for me, which made me feel like I didn’t matter” (Daemen et al. 2025, p.95).   “My uncle and his wife don’t fully understand what my mom is going through… and because of that, we’ve lost that part of the family” (Sparbel et al., 2008, p.332). |
| Emotional and psychological burdens | Distress and overwhelm | Low mood in connection with caregiving.   A diagnosis is life-changing and sparks low mood. | “I was actually diagnosed with depression from all the work, ‘cause I was sleep deprived from staying up with my dad, so…” (Williams et al., 2009, p.7). “Once the diagnosis came through then that just had a tremendous impact on his life, and of course mine… he’s been very depressed” (Dawson et al., 2004, p.126). |
|  | Grief, loss, and mourning | Resentment over loss of childhood.      The emotional pain of mourning the living. | “My father and sister were HD patients. I saw aggression, the desire to kill, they ruined my life, nobody will bring me back my youth ages” (Hubčíková et al., 2022, p.9).   “What hurts me most is that you lose the one you loved” (Røthing et al., 2014, p.702). |
|  | Repression | Avoidance as a useful coping strategy.  Maintenance of psychological wellbeing through avoidance/staying in the present. | “What’s wrong with blocking it out? Maybe that’s what I’ve been doing for the last five years but it’s worked quite well for me” (Maxted et al., 2014, p.342).  “You just deal with whatever comes… if you try and dwell on things too much, I actually think it makes you even worse off… I’d probably have a breakdown” (Brewer et al., 2008, p.12) |
|  | Anxiety and fear | Hypervigilance around symptom onset in the self  Hypervigilance around symptom onset in children | “You forget things or names… it only takes a couple of things to happen… for you to start panicking and think ‘oh my god, is this it, is it starting?’” (Wieringa et al., 2021, p.379). “I used to just sit and watch my kids. One of my kids spilled something, I thought, ‘Oh my God, no’… you know” (Williams et al., 2009, p.794) |
